# A Stakeholder-Engaged Process to Design and Implement the Assessment of Cognitive Complaints Toolkit for Alzheimer’s Disease (ACCT-AD) in Primary Care

**DOI:** 10.21203/rs.3.rs-7134103/v1

**Published:** 2025-07-29

**Authors:** Alissa B. Sideman, Cecilia Alagappan, Ignacia Arteaga, Andrew Breithaupt, Sahar Soleymani, Hrishikesh Belani, Teresa Pham, Sunny Pak, Teresa Sigala, Jason Gravano, Loren Alving, Freddi Segal-Gidan, Howie Rosen

**Affiliations:** University of California; University of California; University of California; University of California; Olive View-UCLA Medical Center; Los Angeles County Ambulatory Care Network; OnLok PACE; OnLok PACE; University of California, San Francisco- Fresno; University of California, San Francisco-Fresno; University of California, San Francisco-Fresno; University of Southern California; University of California

**Keywords:** dementia, diagnosis, primary care, cognitive assessment

## Abstract

**Background:**

Dementia is underdiagnosed, particularly in primary care settings where most people receive their healthcare. These is a need for tools to assist with the diagnosis of dementia by primary care clinicians, who greatly outnumber specialists.

**Objective:**

To describe the collaborative design process, implementation, and lessons learned when developing a new cognitive assessment tool for primary care settings.

**Design and Participants:**

We used an iterative approach to develop, test, and revise the Assessment of Cognitive Complaints Toolkit for Alzheimer’s Disease (ACCT-AD), and used qualitative and survey-based methods to identify lessons learned from its use in four community primary care practices in California

**Key Results:**

Lessons learned from implementing the ACCT-AD toolkit in community primary care practices include the importance of stakeholder engagement in the process, assessing and adapting workflow, staffing, and approach; the educational value of the toolkit as a systematic tool, user response to the toolkit, and challenges around workflow, integration, and sustainability.

**Conclusions:**

The design and implementation of the ACCT-AD toolkit explicitly target workforce constraints that will continue to emerge as demand for cognitive assessment increases. Our approach, which enables primary care clinicians to complete a thorough assessment within their practice, supports building on the strong foundation of the doctor-patient relationship in primary care, and can lead to earlier diagnosis and more efficient referrals.

## Introduction

More than seven million people in the United States live with dementia, and the number is expected to triple in the next thirty years.^1^ The health care system currently provides insufficient services to patients facing cognitive decline and is not prepared to address the need for timely and accurate diagnosis of dementia.^2–5^

Dementia is underdiagnosed, particularly in primary care (PC) settings where most people receive their healthcare.^6–10^ Although primary care clinicians (PCCs) are often the first to see patients with suspected dementia, as many as 66% of patients in PC are not diagnosed in the early stages of the disease.^11–14^ Many clinicians do not pursue formal assessment of cognitive complaints, postponing plans to evaluate them or to refer them to specialists, in part due to resource and time constraints, low confidence, and limited availability of specialists.^5,13,15–19^

The large numbers of patients needing assessment for cognitive complaints can overwhelm specialty practices and referral centers, leading to delays in assessment.^15,20,21^ In one study, the average time between initial symptom recognition and diagnosis was 30 months.^7^ Furthermore, even when a referral for specialty evaluation has been made, less than half are completed.^15^ Diagnostic delays can be reduced if PCCs perform some of the assessment that would be done by specialists.^22^

The diagnosis of dementia relies on recognition of specific symptoms.^23^ Alzheimer’s disease (AD), the most common cause of dementia, is usually characterized primarily by memory loss. However, atypical presentations of dementia are characterized by a variety of non-memory symptoms, including language problems, psychiatric symptoms, changes in personality, or movement problems. A critical aspect of dementia diagnosis and care planning is to assess for the presence of these non-memory features. Under-identification of these symptoms is a key reason that many patients with non-AD dementias are misdiagnosed as having AD or a psychiatric illness.^2,24^ As new treatments targeted at specific etiologies of dementia are approved, this type of error will have increasing significance.^25,26^

These considerations underscore the need for tools to assist with the diagnosis of dementia by PCCs. If PCCs diagnose more patients with the most common form of dementia (AD), and detect features that might suggest atypical causes, this would allow more rapid assessment of cognitive complaints and identification of patients who could be eligible for specific treatments, while facilitating recognition of less common types of dementia that require specialty evaluation.

### The Assessment of Cognitive Complaints Toolkit for Alzheimer’s Disease (ACCT-AD)

In 2018, the ten California Alzheimer’s Disease Centers (CADCs) developed the Assessment of Cognitive Complaints Toolkit for AD (ACCT-AD) to guide PCCs on the history, physical examination, and laboratory and imaging studies for diagnostic evaluation.^27,28^ ACCT-AD was developed by a committee of dementia care experts and takes a unique approach by specifying wording for questions and prompts, and providing interpretation of typical answers encountered in clinical practice as consistent with normal aging, consistent with AD or suggestive of a non-Alzheimer’s dementia. The toolkit also supports identification of features that may indicate a non-degenerative dementia, including potentially treatable etiologies for cognitive impairment, such as mood disorders and sleep apnea. It provides example scripts to facilitate the PCC’s discussion of difficult topics such as diagnostic disclosure, driving evaluation and potential cessation, behavioral symptoms, and participation in research. It also includes guidance for billing for different aspects of the diagnostic workup and chronic care management.

In July of 2019, UCSF received a five-year grant from the California Department of Public Health to support continued development of the toolkit and to pilot its implementation in community PC practice. This project was implemented in collaboration with two other CADCs: the Alzheimer’s and Memory Center at UCSF Fresno, and the USC/Rancho Los Amigos California Alzheimer’s Center in Los Angeles. Our objective in this manuscript is to share our approach and lessons learned in the implementation of this toolkit.

## Methods

### Objectives

**Figure 1:**
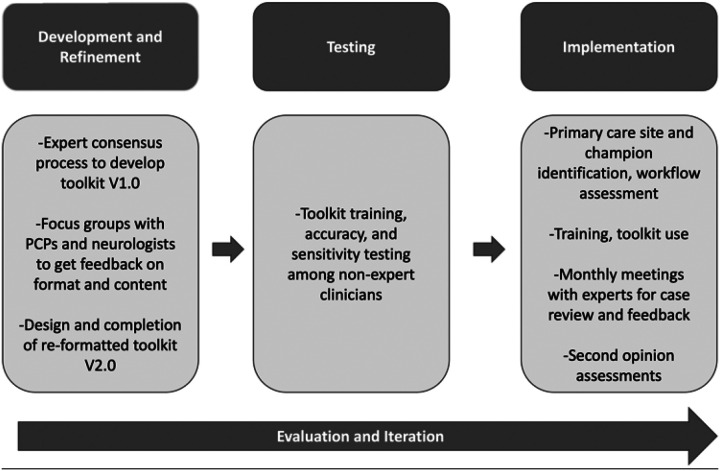
**XXXXX** Legend not included with this version.

Our work was designed to support the development, implementation, and evaluation of the ACCT-AD toolkit and optimize its useability by PCCs in these three phases (Figure 1). Research was approved by the Human Subjects Institutional Review Board at the University of California, San Francisco. All participants provided informed consent.

#### (1) Phase 1: Toolkit development and refinement

##### Focus Groups and Feedback Sessions

We conducted seven focus groups or one-on-one feedback sessions between 2019 and 2022 with PCCs (internal medicine and family medicine, including Medical Doctors (MDs), Nurse Practitioners (NPs), and Doctors of Osteopathy (DOs)) using a focus group guide developed for this study (Supplementary Material 3). Participants included attendings and trainees. Participants received copies of the toolkit ahead of time. The sessions focused on facilitators and barriers to dementia assessment in their practice, and feedback on the toolkit. Participants reported a lack of systematic approach to diagnosing dementia in their clinics and Identified time as the major constraint. They found the toolkit valuable as an educational tool but were concerned about the length and format. They suggested the following modifications: the addition of a self-administered questionnaire, a redesign to make it easier to track patient responses, electronic health record (EHR) integration, and guidance identifying and diagnosing patients with Mild Cognitive Impairment (MCI).

##### Toolkit V2.0

In response to this feedback, as well as ongoing input during the accuracy-testing and PCC recruitment phases, we developed ACCT-AD Toolkit V2.0, (Supplemental Material 1 and 2), which includes several modifications: 1) a revised format that improves usability by dividing the toolkit into three components: the instructions, forms that can be used for documentation of findings from an assessment, and a reference manual for use in interpreting findings, 2) additional materials to support diagnosis of Mild Cognitive Impairment; 3) smart phrases that can be used to document the assessment in the EHR, 4) a pre-visit questionnaire that can be implemented over the phone or online with the patient and a knowledgeable informant prior to the visit; this questionnaire uses simple language to collect the same information as the in-person toolkit, and has been translated into Chinese, Vietnamese, and Spanish. The goal of the pre-visit questionnaire is to streamline the clinician’s in-person work by providing responses to questions in advance. However, because of concerns that the sensitivity of the questions might vary across languages and cultures, we cautioned practices that self-reported responses should be reviewed in person by an experienced member of the practice to ensure that the responses were addressing the area of concern targeted by the question. We also added several educational components related to mild cognitive impairment, vascular cognitive impairment, biomarkers for neurodegenerative disease, and genetic testing for neurodegenerative disease (NDD).

#### (2) Phase 2: Initial implementation

We used the training infrastructure of the California Alzheimer’s Disease Centers (CADCs) to provide initial feedback on the use of the toolkit in practice. Each of the CADCs has relationships with local healthcare institutions that have clinical training programs. At UCSF, the Memory and Aging Center conducts a four-week rotation that trains UCSF neurology and psychiatry residents, geriatrics fellows, and medical students. The UCSF-affiliated program in Fresno trains family medicine residents, and the CADC at Rancho Los Amigos, which is affiliated with the University of Southern California, trains family medicine residents from the Charles Drew University associated with Martin Luther King Jr. Hospital. Trainees independently collect the history and physical examination and other data, and then present this information to the attending physician, who provides the final diagnostic assessment. For this project, we asked these trainees to use the toolkit for their assessments and to provide feedback about the toolkit. During the project period, 102 trainees used the toolkit. This phase was utilized to make adjustments to the diagnostic guidance and wording of individual questions as needed.

#### (3) Phase 3: Implementation in community practice

##### Champion identification and workflow assessment

We began implementing the toolkit as a quality improvement tool at four community practice sites: Olive View Medical Center in the Los Angeles County Health System, the Central California Faculty Medical Group (CCFMG) in Fresno, Martin Luther King Jr. Hospital in Los Angeles, and the On-Lok Program for All-Inclusive Care for the Elderly (PACE), in the San Francisco Bay Area. These sites were Identified based on existing relationships with the study PIs and interest from stakeholders in expanding dementia assessment in their settings. Prior to implementation, two investigators conducted multi-day site visits to meet with key stakeholders and assess existing clinic workflow to identify ways to best fit the toolkit within existing practices. The implementation approach at each site varied based on clinic workflow, but each site was required to retain fidelity to the questions and diagnostic pathway recommended in the toolkit. Clinic directors served as the main liaison between the primary care clinic and the study leadership. They devised a strategy to introduce the toolkit to PCCs within their clinic and met regularly with the study leadership to advise on revisions to the toolkit and study procedures. Individual PCCs were invited to participate in this project on a voluntary basis, and were not given any special accommodations, such as changes in their patient load or schedule. The clinics received funding from the grant to support clinic leadership to meet regularly with the other study personnel to review study progress, and to support staff to facilitate entry of data from the clinic evaluations into the study database. Individual PCCs received small gift cards for entering data into the study database and completing a brief survey developed for this study (Supplementary Material 4).

##### Training

Each PCC participated in a 1-hour training presentation on the toolkit. PCCs were also given access to the Montreal Cognitive Assessment (MoCA) training certification program. After receiving feedback from community practitioner stakeholders that it would be helpful to have a regular meeting with neurology experts to discuss toolkit implementation, build community with other sites, present cases, and get insights about challenging cases, we implemented monthly all-site meetings where PCCs presented patient cases they assessed using the Toolkit. Sites reported benefitting from hearing implementation approaches from the other sites involved in the study and getting expert input on challenging cases.

##### Monthly meetings, feedback sessions, surveys, and interviews

The monthly all-site meetings included feedback from the PCCs, who suggested potential improvements. We also implemented pre/post surveys about PCC practices, attitudes, and challenges doing dementia diagnosis and care and pre/post interviews or focus groups with PCCs enrolled in the study about approaches to dementia diagnosis, facilitators and barriers to dementia diagnosis and care, training and educational needs, and experiences using the toolkit. We are engaged in ongoing analysis of the information gained from Phase 3 to evaluate and iterate upon the toolkit as needed and to identify common challenges that need to be addressed before more widespread implementation is possible.

##### Community practice accuracy testing

A small subset of individuals evaluated in PC also had a second opinion assessment at one of the three expert centers involved in the project. We assessed PCC diagnostic accuracy for these patients by comparing PCC’s diagnosis with that of the expert.

## Results

### Implementation Outcomes

**Figure 2:**
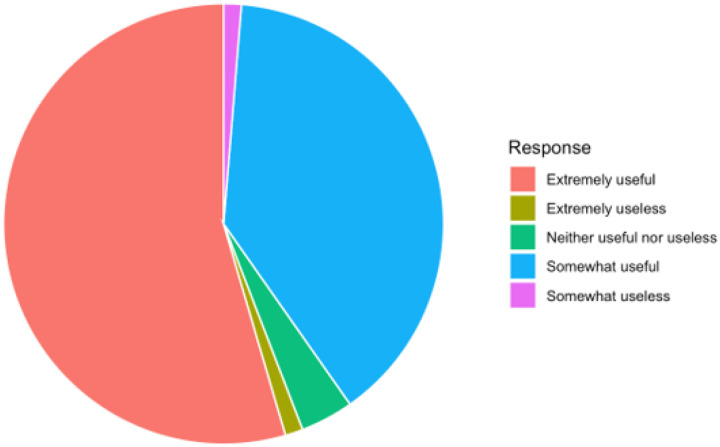
How useful was the ACCT-AD Toolkit for identifying a probable diagnosis?

Between July 2019 and July 2024, the toolkit was used in the PC setting by 22 clinicians (Table 1) to make etiological diagnoses for 137 cases. The toolkit was rated by PCCs as being extremely helpful or somewhat helpful in 94% of the cases (Figure 2), and nearly all felt confident in their diagnosis. Patient demographics and language of assessment are summarized in Table 2. Diagnoses were dementia in 29%, MCI in 47%, and no significant cognitive impairment in 23% (Table 3). Features suggesting atypical neurodegeneration in 14 patients are listed in Table 4. Symptoms were thought to be due to non-neurodegenerative conditions in 35% (most with MCI), and the conditions thought to be contributing are listed in Table 5.

### Lessons Learned

The project successfully implemented this toolkit in four busy PC practices over the course of 5 years. We found that implementation was feasible within PC even though it required additional training and organization of time for assessment that was not always easily available in PC. The lessons, summarized below represent key factors that influenced the success of implementation (Table 6).

#### Lesson 1: Stakeholder engagement.

Identification of implementation champions and engaging in iterative stakeholder engagement were critical. PCCs were involved throughout the process in giving us feedback. Many of our champions were clinic directors. We found that the clinic director’s interest and cooperation fostered willingness and greater engagement with the new tool among other clinicians in the practice.

#### Lesson 2: Targeted and responsive educational support during implementation.

The need to reinforce the recommended diagnostic approach with changes and additional training quickly became apparent when we found during early, informal case reviews that some PCCs had difficulty separating the severity of the impairment from the potential etiology. PCCs would diagnose patients with dementia when the symptoms suggested AD, even if the severity of the impairment was consistent with mild cognitive impairment (MCI). This prompted a revision of the toolkit to create a new section discussing MCI. In addition, we created a monthly case conference to reinforce the principles of interpretation being promoted in the toolkit. Using a standard template, PCCs presented cases they had assessed in their clinics at meetings that included experts and other PC users. These meetings served to check that the toolkit was guiding appropriate decision-making while educating all users.

#### Lesson 3: Working with clinics to identify how to fit the approach to their workflow

##### 3a. Assessing and adapting workflow and staffing.

We found that it was important to support clinic-specific strategies for toolkit implementation. At the start of the study, two researchers did site visits to learn about each site’s workflow and meet with the clinic directors. In some settings, clinic staff or social workers were able to collect pre-visit questionnaires, a model that made it easier to support the PCC during the in-person visit. Some clinicians made adjustments to see patients across multiple visits to complete the toolkit, while others engaged clinical staff to assist with aspects of the toolkit.

##### 3b: Adaptation of cognitive testing.

Although the toolkit originally focused on the MoCA, sites also Identified tests that were already being used in practice. They highlighted their need to assess patients with low education and who did not speak English. We incorporated additional options for cognitive testing into the toolkit instructions and provided guidance on versions of these tests that had been validated in specific languages and in cohorts with low levels of education. We provided education-specific thresholds to assist with diagnostic assessments.

#### Lesson 4: Value of toolkit

##### 4a. Educational value of the toolkit.

PCCs Identified many aspects of the toolkit that they found valuable. Many felt the toolkit was useful as an educational tool, helping them think more about differential diagnosis, and whether the problem is normal aging. They felt the toolkit helped them to ask better questions and learn how certain questions and answers pertain to dementia. The toolkit was enthusiastically adopted by one of the sites as a training for family medicine residents.

##### 4b. Usefulness of a systematic tool.

PCCs also reported that the toolkit created a systematic approach for knowing what to address with patients while also guiding their diagnostic interpretation. They felt it was helpful to have specific questions and a framework that links specific responses to diagnosis, and they appreciated integration of caregivers.

##### 4c. Patient appreciation of in-depth assessment and education.

PCCs also noted that patients and families with whom they had used the toolkit felt that their assessment was thorough, and it created peace of mind for the “worried well”. One PCC reported, “It helped me learn to know what kind of questions to ask and to get to know the patient and their families better.”

##### 4d. Support for a team-based approach.

PCCs also appreciated the integration of other clinical team members who could support the diagnostic process. In particular, the pre-visit questionnaire could be administered to patients and care partners by other team members ahead of time, freeing valuable time for PCCs and streamlining the visit because they knew which questions to focus on.

#### Lesson 5: Challenges around workflow, integration, and sustainability

We also Identified several challenges in the implementation of the toolkit, many related to known barriers in primary care related to time and staffing constraints. PCCs Identified the learning curve for use of the toolkit, which takes an investment of time upfront until they are comfortable. Furthermore, practitioners found it challenging to follow up with next steps of the diagnostic process, such as disclosure, treatment recommendations (if any), and referrals to specialist settings, if needed, because of time constraints. The toolkit was best used in a team-based approach, where a staff member in the clinic implemented the pre-visit questionnaire ahead of time, but this required identifying a person in the clinic. In one clinic, this was a medical assistant. However, not all clinics have access to sufficient staffing. At another clinic, the pre-visit questionnaire data was delivered by a non-professional, trained coordinator supported by the research grant. The training and educational background for this individual was similar to that of patient care navigators that have been used for multidisciplinary dementia care models. Some PCCs felt it was challenging to set up visits with patients, as sometimes the toolkit took multiple visits to implement. Those who did not have it incorporated into the EHR felt that it was not streamlined and could have been easier to use if it was integrated, which was done at some sites based on user feedback.

## Discussion and Next Steps

We have described our stakeholder-engaged approach to building a toolkit to support community PCCs in evaluating cognitive and behavioral changes that could represent typical, late-onset dementia due to Alzheimer’s disease. Earlier identification of dementia can lead to faster implementation of management, including treatment of reversible conditions, referrals to specialists in whom this is appropriate, identification of supportive services, and critical planning for those who have mild cognitive changes while they still have capacity. Timely diagnosis has also been associated with reduced costs for healthcare services.^2^ As targeted treatments that rely on a specific diagnosis become available, earlier and more accurate diagnosis will become increasingly important.^25,26^

Our initial experience with the ACCT-AD toolkit indicates that several components are instrumental for implementation in primary care. The first of these is concrete guidance. Clinical guidelines recommend collecting a patient history, laboratory workup, cognitive screening, and imaging, and often include brief descriptions of atypical dementias. Rarely do they provide guidance on how to collect this history from both the patient and an informant and how to interpret cognitive testing.^16,29,30^ We found that PCCs appreciated specific guidance on what questions must be asked, how anticipated responses should be interpreted, and how the history should be integrated with other data to guide diagnosis. Stakeholder engagement was another key element for success. Partnerships between dementia experts and PCCs were utilized to test and amend the toolkit in a user-centered way while maintaining the best-practice principles of dementia diagnosis. Engaging stakeholders early and often in building tools that are feasible in their practice settings is important to set the stage for the rollout of treatment and care innovations.^31,32^ Such engagement will continue to be needed as the field rapidly evolves to incorporate new biomarkers and treatments.^22,33,34^

The design and implementation of the toolkit explicitly target workforce constraints that will continue to emerge as demand for cognitive assessment increases.^22^ Our approach, which enables PCCs to complete a thorough assessment within their practice supports more efficient referrals, including identifying patients that may not need a referral to dementia specialists. For example, 40 percent of the cases evaluated in this project were thought to be cognitively normal or were Identified as having a non-neurodegenerative disorder that needed to be addressed. For those referred to dementia specialist, the assessment supported by the toolkit ensures that the evaluation up to that point has been complete, making the questions that need to be addressed by the specialist more focused. Use of this toolkit therefore represents a strategy to improve the efficiency of the health system in the evaluation of persons with cognitive changes.

Use of this toolkit can be considered as part of a larger strategy to create networks of dementia-proficient community practices through programs such as Project ECHO for dementia, which supports dementia diagnosis and care through case conferences similar to those implemented for this project.^35^ A shared diagnostic framework, implemented through a common instrument that PCCs endorse, can provide the foundation for such networks. Common approaches for assessment would improve communication among clinicians and with patients and set the stage for rapidly disseminating new developments that affect diagnosis and care for neurodegenerative diseases. As potential treatments and options for diagnostic testing evolve, this network can serve as a resource for disseminating knowledge. This workforce innovation has the potential to improve health service delivery for people with dementia.

### Limitations

Although the results of this pilot program are encouraging, there are many outstanding questions. Most patients seen in PC for this project were not evaluated by experts, so that the accuracy of PCC diagnoses could not be determined for most of the patients. Accuracy of diagnosis using this tool might vary considerably depending on the experience of the clinician and patient factors. In addition, although the pre-visit questionnaire can potentially improve efficiency of assessment, the sensitivity and specificity of these questions, particularly in diverse clinical settings, has not been established, so that review by a trained staff member is required. These issues will be addressed in a project recently funded by the National Institute on Aging (1R01AG085689–01A1).

## Supplementary Material

Supplementary Files

This is a list of supplementary files associated with this preprint. Click to download.

- SupplementaryMaterial1ToolkitQuestionnaire.pdf
- SupplementaryMaterial2ToolkitInterpretationManual.pdf
- SupplementaryMaterial3FocusGroupGuide.docx
- SupplementaryMaterial4QualtricsSurvey.pdf

## Figures and Tables

**Table 1.** Clinicians Using the Toolkit in Primary Care Practice

Site	Number of Clinicians	Number of Patients
Olive View	11 (X MD, X NP)	99
CCFMG	1 MD	26
On Lok	10 (X MD, X NP)	12

**Table 2.** Demographics for 137 Cases Diagnosed in Primary Care

Age		Education		Language of Assessment	
>65	67%	College or more	25%	Spanish	56%
<65	33%	High School	23%	English	23%
		Middle School	13%	Cantonese	6%
		Elementary School	32%	Mandarin	2%
		No Formal Education	7%	Other	3%

**Table 3.** Diagnoses for 137 Cases Diagnosed in Primary Care

Syndrome	N (%)	Suspected Cause	N (%)
Dementia			
	40 (29%)	Typical AD	23 (58%)
		Atypical Neurodegenerative	14 (35%)
		Non-Degenerative	8%
		Not enough symptoms to choose	0
Mild Cognitive Impairment			
	65 (47%)	Typical AD	3 (5%)
		Atypical Neurodegenerative	0
		Non-Degenerative	45 (69%)
		Not enough symptoms to choose	18 (28%)
No Significant Cognitive Impairment			
	32 (23%)		

**Table 4.** Atypical Features in 14 Patients with Atypical Neurodegenerative Syndromes

Symptoms Not Typically Seen in Alzheimer’s Disease	21%
Early Age of Onset	14%
Rapidly Progressive	14%
Motor Features	16%
Not specified	44%

**Table 5.** Identified Conditions in 48 Patients with Non-Neurodegenerative Syndromes

Depression or Other Psychiatric Condition	75%
Sleep Apnea	35%
Cerebrovascular Changes	17%
Hearing or Visual Impairment	16%
Laboratory Abnormalities	6%

**Table 6.** Lessons Learned

**1.** Stakeholder engagement
**2.** Targeted and responsive educational support during implementation
**3.** Working with clinics to identify how to fit the approach to their workflow
**3a.** Assessing and adapting workflow and staffing
**3b.** Adaptation of cognitive testing.
**4.** Value of the toolkit
**4a.** Educational value
**4b.** Usefulness as a systematic tool
**4c.** Patient appreciation of in-depth assessment and education
**4d.** Support for a team-based approach
**5.** Challenges around workflow, integration, and sustainability

## Data Availability

The datasets used and/or analyzed during the current study are available from the corresponding author on reasonable request.
